# Delta large-channel technique versus microscopy-assisted laminar fenestration decompression for lumbar spinal stenosis: a one-year prospective cohort study

**DOI:** 10.1186/s12891-023-06143-0

**Published:** 2023-01-19

**Authors:** Yuehang Sheng, Jing Li, Lei Chen, Minghao Geng, Jing Fen, Shaodong Sun, Jianhua Sun

**Affiliations:** Department of Spinal Surgery, The First Affiliated Hospital of Shihezi University School of Medicine, Shihezi, 832000 China

**Keywords:** Lumbar spinal stenosis, Spinal endoscopic techniques, Microscopy, Clinical outcomes

## Abstract

**Purpose:**

When it comes to treating lumbar spinal stenosis (LSS), a procedure known as microscope-assisted fenestration decompression has expediently become the gold standard. With the advancement of spinal endoscopy, the Delta large-channel approach has shown promising clinical outcomes in the management of lumbar spinal stenosis. However, case studies of this method being used to treat lumbar spinal stenosis are still uncommon. The purpose of this research was to examine how well microscopy-assisted laminectomy and the Delta large-channel approach work in treating LSS in the clinic.

**Methods:**

From May 2018 to June 2020, 149 patients diagnosed with LSS were divided into 80 patients in Delta large-channel technique groups (FE group) and 69 patients in microscope groups (Micro group). Lower back and lower limb pain were measured using the visual analogue scale (VAS-LBP and VAS-LP), while lower limb numbness was evaluated using the 11-point numerical rating scale (NRS-LN); modified Oswestry Disability Index (ODI) was used to evaluate the quality of life, and modified MacNab criteria were used to assess the clinical efficacy before surgery and at one week, three months, six months, and 12 months after surgery. All patients had single-level lumbar spinal stenosis, and clinical data such as hospital stay, operation time, intraoperative blood loss were statistically analyzed.

**Results:**

Finally, 111 patients (62 in FE group and 49 in Micro group) completed follow-up. Compared with preoperative results, postoperative VAS-LBP, VAS-LP, NRS-LN score and modified ODI score were significantly improved in 2 groups (*P* < 0.05), but there was no significant difference in postoperative follow-up at each time point (*P* > 0.05), Except 1 week after surgery, VAS-LBP in FE group was lower than that in Micro group (*P* < 0.05). It is noteworthy that the FE group had a shorter hospital stay, less intraoperative blood loss, and a quicker time of getting out of bed when compared with the microscope group，but the operation time was just the opposite (*P* < 0.05). The excellent and good rate was 83.87% in FE group and 85.71% in Micro group (*P* > 0.05).

**Conclusions:**

Both microscope-assisted laminar fenestration decompression and Delta large-channel procedures provide satisfactory treatment outcomes, however the Delta large-channel approach has some potential advantages for the treatment of LSS, including quicker recovery and sooner reduced VAS-LBP. Long-term consequences, however, will necessitate additional follow-up and research.

## Introduction

Low back pain and sciatica, with or without neurogenic claudication and cauda equina syndrome, are the most prevalent symptoms of lumbar spinal stenosis (LSS), a common degenerative condition originally clinically documented in 1954 by a Dutch neurosurgeon dubbed Henk Verbiest [1]. The central canal, lateral recess, and neural foramen are all potential locations for lesions in LSS [2]. Age-related alterations to the spine are responsible for the majority of degenerative LSS. During degeneration, the disc, ligament flavum, and facet joints are transformed, ultimately decreasing the accessible space for spinal nerves and blood vessels [3, 4]. The prevalence of LSS is about 9.3%, prevalent in people over 60 years of age [5] and maybe as high as 80% in specific populations [6], of which 30% of patients may present with severe lumbar stenosis symptoms. Approximately 17% of patients suffer from long-term intermittent neurogenic claudication, drastically diminishing the quality of life of these patients [7].

Conservative treatment is preferred for LSS, and patients usually require 4 to 6 weeks of physical therapy [8]. If conservative measures fail to alleviate a patient’s LSS symptoms, surgery may be an option [9]. Open laminectomy is the most common kind of surgery used to treat LSS, and it is also the most conventional method [10, 11]. This device, however, is significantly traumatizing, resulting in extensive bone destruction as well as damage to the paravertebral muscles and ligaments, thus leading to low back pain and postoperative low back syndrome. It is also suspected to be a contributing factor to iatrogenic instability [12–15]. The development of minimally invasive spinal techniques has been a successful solution to this problem. The procedure is widely accepted by surgeons because of its long-term follow-up efficacy, which makes it the most commonly used and effective for treating lumbar spinal stenosis [16]. However, Elderly and some medically ill patients with lumbar spinal stenosis continue to face challenges when it comes to treatment [17].

The use of Delta large-channel technology for the treatment of LSS is currently in its infancy. It remains unclear whether either of the above methods is safe and effective for treating LSS; few studies have compared them [18]. Our pilot study compared the Delta large-channel technique with microscope-assisted laminar fenestration in treating LSS with the objective of comparing their effectiveness.

## Materials and methods

It is important to note that all patients provided written informed consent before participating in the study, and all experiments were performed according to the relevant specifications after obtaining consent from the hospital ethics committee. A prospective analysis was carried out on 149 patients who underwent lumbar decompression surgery between May 2018 and June 2020 by a single physician at the same institution. The patients were carefully included and excluded according to strict inclusion and exclusion criteria. Finally, 111 completed the follow-up (follow-up rate 74.50%), of which 62 underwent total endoscopic decompression (FE group), and 49 underwent microscopic decompression (microscopic group). The mean follow-up time was 13.3 ± 4.3 months.

Inclusion criteria (1) Low back pain with unilateral radiating pain and/or numbness in the lower extremities; (2) A physical examination confirms the symptoms of the illness and there is no difference in the duties; (3) A series of imaging examinations, including X-rays, CTs, MRIs, and other forms of imaging, indicated that the segments responsible were consistent with the symptoms and signs observed; (4) Conservative treatment failed for 3 months or symptoms worsened; (5) Sign informed consent. Exclusion criteria: (1) Concurrent cervical or thoracic decompression or multilevel lumbar stenosis; (2) With vertebral fracture, intravertebral infection or tumor; (3) Previous lumbar surgery; (4) Lumbar scoliosis > 20°; (5) Lumbar spondylolisthesis grade I or above; (6) Patients who cannot understand the details of the study.

### Image

X-rays, CT scans, and magnetic resonance imaging were all taken of all patients’ lumbar region (MRI 1.5 T). Lumbar instability and spondylolisthesis were examined by anteroposterior and lateral radiographs and flexion and extension radiographs. The calcification of ligamentum flavum and herniation of the disc of the lumbar spine were observed using CT images of the spine. Lumbar MRI is used to observe the severity of lumbar spinal stenosis.

### Assessment outcome measures

A visual analogue scale (VAS: 0: no pain, 10: worst pain) was used to assess the degree of low back pain and lower limb pain. An 11-point numerical rating scale (NRS; 0: no numbness, 10: most severe numbness) was used to evaluate the degree of numbness in the lower extremities. The modified Oswestry Disability Index (ODI; from 0 to 100%, with more severe disability) score and postoperative lumbar function was assessed by the modified MacNab criteria (excellent, good, fair, and poor). All data were collected by patient self-report. Clinical data included operation time, intraoperative blood loss, and hospital stay.

### Surgical method

The surgeon makes an all-encompassing decision on the surgical approach to take based on the patient’s condition. In this study, both Delta large-channel technique and microscope-assisted laminar fenestration were inpatient procedures and performed under general anaesthesia and endotracheal intubation. In terms of postoperative analgesia, there were no differences between the two groups.

Microscope-assisted vertebral plate fenestration:(1) The patient was completely anesthetized and in the prone arch bridge position; (2) Positioning duties of Kirschner needle and G-arm machine; (3) Take the lesion as the center and move to responsible end to 3 mm The paraspinal muscle was separated from the deep fascia about 4 cm longitudinally, the working channel was expanded, the surgical field of view was fully exposed, and the attached muscle tissue of the lumbar lamina was stripped under the microscope, the residual soft tissue outside the lamina was removed, and the bleeding was stopped. The intervertebral space was gradually polished using high-speed drilling to preserve as much bone as possible from the dorsal lamina. (4) Exfoliating the thickened ligamentum flavum, exposing the spinal canal and lateral recess, and releasing the lateral nerve roots were some of the procedures performed during this operation.(5) The operating table should be tilted about 30°. Use a bone masher or a small drill to decompress the contralateral recess of the facet joint; (6) An indwelling of negative pressure drainage was performed on the wound, which was thoroughly hemostasised as well as cleaned. Layers of the operation were carried out layer by layer.

**Fig. 1:**
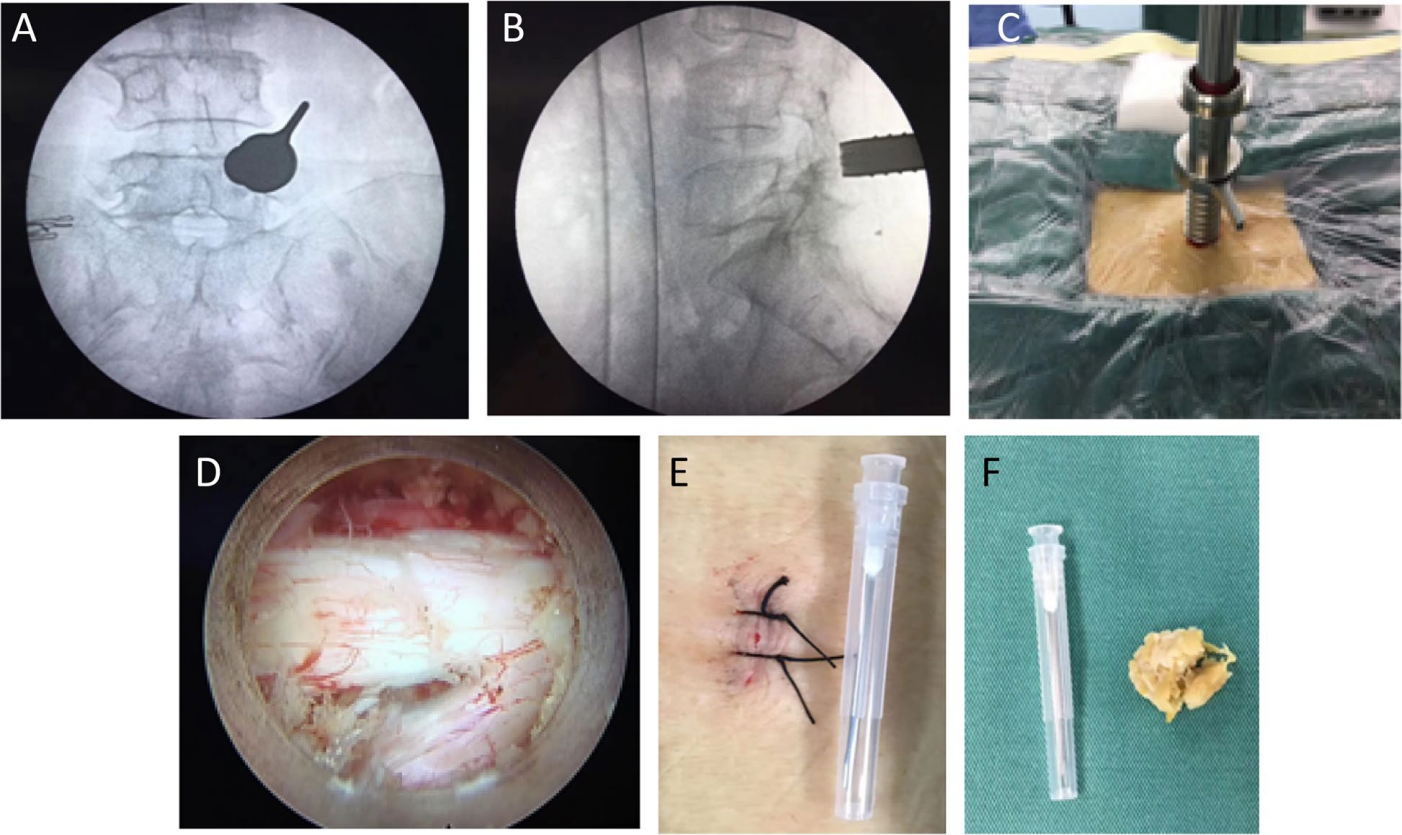
**AB** Responsibility phase localization assisted by G-arm machine; **C** Intraoperative Delta large channel step-by-step working cannula; **D** Display decompressed and relaxed nerve roots under endoscope screen; **E** Length of surgical incision; **F** Hypertrophic ligamentum flavum tissue removed during surgery

Delta large-channel technique: (1) As part of the anesthesia process, the patient was placed in a prone arch bridge position and anesthesia was administered fully; (2) It was performed with a Kirschner needle in vitro and the tip was located in the intervertebral space responsible for the condition by G-arm fluoroscopy. A puncture point, 0.5 cm above the spinous process on the pathological side, was selected and marked externally on the upper edge of the intervertebral foramen; (3) Routine disinfection and towel laying; (4) Approximately one centimeter of longitudinal incision was made at the center of the puncture point in order to gradually insert the dilator tube, along with the Delta working cannula, and to further position the intervertebral space at this location using fluoroscopic guidance on the G-arm (Fig. 1AB). (5) Having determined the location, it is necessary to extract the knot from the guide bar, to connect the light source to the camera, to turn on the light source, to adjust the white balance as well as the amount of water to be used (Fig. 1C); (6) Clean the soft tissue attached to the surface of the laminae, expose the foramina intervertebral, along the lower margin of the articular process of the upper vertebral body and the upper margin of the articular process of the lower vertebral body, bit off the bone about 1 mm, expand the osseous laminae space, expose the origin and stop of the ligamenta flavum respectively; It was necessary to remove the ligamenta flavum in its entirety. A thorough exposure of the dura mater and nerve roots was achieved by resection of the residual ligamenta flavum and the cohesive part of the hyperplasia of the articular process. (7) The dura mater and the nerve root were carefully separated using a nerve stripper and a tube in tube kit, and the nerve root and the dura mater were pulled to the contralateral side to expose the fibrous ring. Herniation of the disc and intact annulus fibrosus were indications that the nerve root tension was high, but the annulus fibrosus tension was maximal at the fixed point if disc herniation was obvious. In order to solidify the nucleus pulposus and annulus fibrosus in the disc, the ball radio-frequency cutter head was used to cut open the annulus fibrosus, remove the internal disc loosening, and remove the free nucleus pulposus. Solidification of the annulus’ surface can be done using a spherical radiofrequency cutter head if the annulus’ surface is intact. An intervertebral disc that is herniated with a rupture of the annulus fibrosus will have the free nucleus pulposus removed first, followed by a retraction of the annulus fibrosus. By solidifying the nucleus pulposus and annulus fibrosus ruptures in the intervertebral disc after the free nucleus pulposus has been removed, the nucleus pulposus and annulus fibrosus are removed. (8) Reexamine the nerve root compression and determine whether the dorsal and ventral sides are visibly compressed. When the tension and relaxation techniques were finally perfected, the pain subsided and comprehensive hemostasis was achieved (Fig. 1D); (9) The working cannula was pulled out layer by layer, the incision was closed layer by layer, the skin was disinfected again, alcohol dressing was performed, and the specimens were removed and sent for pathological examination. The operation is complete (Fig. 1ef).

### After surgery

To treat symptomatic analgesia, all patients were given non-steroidal anti-inflammatory drugs orally postoperatively. In accordance with the drainage volume, time was determined for removing the drainage tube. In the following four weeks following the operation, the waist circumference was worned.

### Statistical analysis

All statistical testing was performed in SPSS, version 20.0. If the quantitative data followed assumptions of normality and homogeneity of variance, the t-test was utilised to conduct statistical analysis; otherwise, the mean and standard deviation were calculated manually; In cases where the data did not fit the assumptions of normality and homogeneity of variance, the Mann-Whitney U test was used to determine a median value (interquartile range); Data from continuous measures were reported as means and standard deviations after being evaluated using a two-way repeated-measures analysis of variance; enumeration data were analyzed by chi-square test, and ranked data were analyzed by Ridit analysis.α = 0.05 was taken as the test level, and *P* < 0.05 were considered to be different.

## Results

Table 1 displays demographic and clinical information for all patients. The mean age of the FE group was 65.74 ± 11.19 (range, 39–84) years and 64.06 ± 9.01 (range, 47–82) years. There were no significant differences between the two groups in baseline demographics (*P* > 0.05), such as age, gender, BMI, responsibility, and medical conditions.

**Table 1:** Baseline characteristics of included patients

Characteristics	EF Group(*n* = 62)	Micro group(*n* = 49)	*P*
Age (years)	65.74 ± 11.19	64.06 ± 9.01	0.395
Gender (*n*/%)			0.852
male	28	23	
female	34	26	
Body mass index	26.02 ± 2.58	25.33 ± 1.04	0.083
Procedure Location			0.599
L2/3	1	2	
L3/4	4	6	
L4/5	52	37	
L5/S1	5	4	
Extent of stenosis			0.459
B	5	7	
C	49	38	
D	8	4	
Concomitant disease			0.930
hypertension	26	21	
coronary disease	6	4	
diabetes	11	8	
cerebral infarction	3	1	
arthritis deformans	3	4	
osteoarthritis	11	7	

The perioperative and postoperative complications were shown in Table 2. The mean operating time of FE group was 92.50 (84.75,97.00) min, which was lower than that of Micro group (75.00 (69.00,78.00) min, and the difference was statistically significant (*P* < 0.05). In the FE group, the average length of hospitalisation was 4 days (3–5 days), whereas in the Micro group, it was 7 days (6–8 days); the intraoperative blood loss was 17.50 (14.00, 25.00) days in the FE group and 125.00 (110.00, 130.00) ml in Micro group; the postoperative off-bed time was 2.00 (1.50, 4.00) days in FE group and 5.00 (4.00, 7.00) days in Micro group; the intraoperative blood loss, hospital stay and postoperative off-bed time in EF group were lower than that in the microscopic group (*P* < 0.05). Despite the fact that there was 1 case of surgical incision infection in each group treated with the second debridement, there was no discernible difference in complications between the two groups (*P* > 0.05). There were three instances of dural tears in FE group and 1 case in Micro group, however the tears were all smaller than 0.6 cm, therefore they were cured after conservative treatment, such as bed rest with occipital removal. Following resting in bed, three patients who had had brief delirium after surgery were able to make a full recovery.

**Table 2:** Perioperative and postoperative complications

Characteristics	EF Group(*n* = 62)	Micro Group(*n* = 49)	*P*
Operation time (min)	92.50 (84.75,97.00)	75.00 (69.00,78.00)	<0.001
Days of hospitalization (days)	4.00 (3.00,5.00)	7.00 (6.00,8.00)	<0.001
Inoperative bleeding volume (ml)	17.50 (14.00,25.00)	125.00 (110.00,130.00)	<0.001
Time to the ground (days)	2.00(1.50,4.00)	5.00 (4.00,7.00)	<0.001
Complications	8 (12.90%)	6 (12.24%)	0.880
Laceration of bursa	3 (4.83%)	1 (2.04%)	
Urinary storage	3 (4.83%)	2 (4.08%)	
Transient delirium	1 (1.61%)	2 (4.08%)	
Operative area infection	1 (1.61%)	1 (2.04%)	

**Fig. 2:**
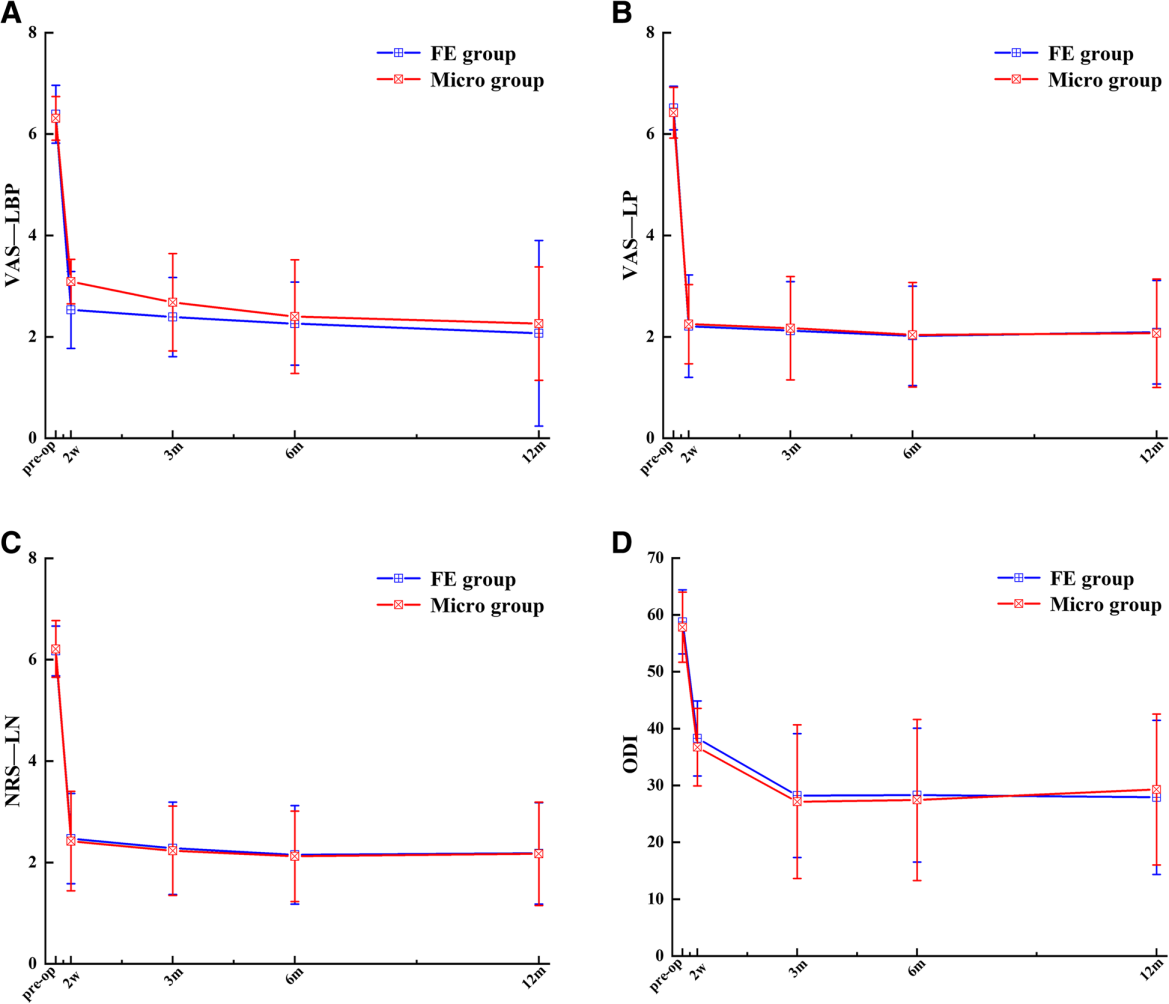
demonstrates the clinical outcomes of patients in the two groups before surgery, 1 week, 3 months, 6 months and 12 months after surgery. (**A**) VAS back pain score; (**B**) VAS leg pain score; (**C**) NRS leg numbness score; (**D**) Oswestry Disability Index (ODI). All groups exhibited substantial variations between pre- and post-operative follow-up indexes (*P* < 0.05). One week after surgery, VAS-LBP in FE group was lower than that in Micro group (*p* > 0.05). No statistically difference was discovered in follow-up indexes among other postoperative groups (*P* > 0.05)

VAS-LBP scores: decreased from 6.42 ± 0.67 to 1.98 ± 1.55 (*P* < 0.05) in the FE group; and from 6.19 ± 0.75 to 2.11 ± 1.62 (*P* < 0.05, Fig. 2A) in the microscopic group. Compared to the FE group, the microscopic group had a reduction in VAS-LP score from 6.09 ± 0.84 to 1.82 ± 1.19 (*P* < 0.05, Fig. 2B), whereas the FE group witnessed a delince from 5.95 ± 0.87 to 2.07 ± 1.93 (*P* < 0.05). In terms of lower extremity numbness, the NRS score decreased from 5.87 ± 0.62 to 2.01 ± 1.31 in the FE group (*P* < 0.05); it fell from 6.02 ± 0.43 to 1.91 ± 1.55 in the microscopic group (*P* < 0.05, Fig. 2C). The FE group had a reduction in their mean modified ODI score, from 58.86 ± 5.64 to 27.90 ± 13.54 (*P* < 0.05); it fell from 57.82 ± 6.16 to 29.29 ± 13.28 in the microscopic group (*P* < 0.05, Fig. 2D). Both preoperatively and at 1, 3, 6, and 12 months postoperatively, there were no statistically significant differences between the two groups in VAS-LBP, VAS-LP, NRS, and modified ODI (*P* > 0.05). In contrast, one week after surgery, VAS-LBP was lower in the FE group than in the Mirco group (2.80 ± 0.92 vs 3.40 ± 0.50, *P* < 0.05).

**Fig. 3:**
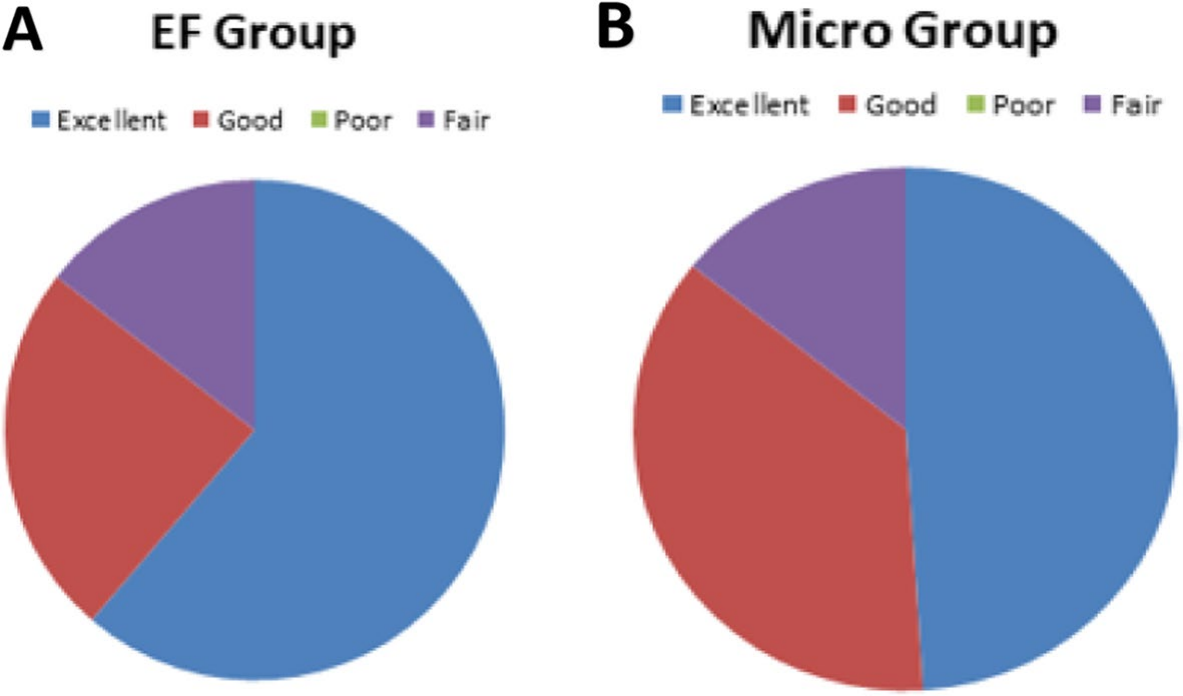
**AB** The patients in the two groups were followed up according to the modified MacNab criteria at 12 months after the operation, in which the excellent rate was 83.87% in the FE group and 85.71% in the microscope group, with no statistical difference between the two groups (*P* > 0.05)

The modified MacNab is depicted in Fig. 3. A total of 83.87% in the FE group (Fig. 3A) and 85.71% in the Micro group achieved an outstanding rating during follow-up (Fig. 3B) (*P* > 0.05).

## Discussion

Good outcomes have been demonstrated using microscope-assisted laminar fenestration decompression for the treatment of LSS, and endoscopic surgery is growing in favor among spine surgeons and patients with LSS as technology and treatment approaches evolve [19–21].

Results showed that both postoperative VAS-LBP and VAS-LP scores were considerably diminished after surgery compared to pre-operative levels; This performance was in keeping with evidence from the prior literatures, and the ODI score further declined considerably throughout follow-up, as had been previously documented [22–24]. Although microscopically fenestration of the lamina is effective and minimally invasive, traditional surgical methods still require the use of a retractor to pull the soft tissue and separate the paravertebral muscles, which is still invasive to the patient and may result in iatrogenic spinal instability. However, low back pain is usually residual after these complications have occurred [18, 20, 23]. In comparison to the microscope-assisted lamina fenestration decompression technique, the Delta large channel technique has the benefits of a smaller surgical incision (< 10 mm) and the working channel. The tubular channel is utilized for orderly tissue dilation, allowing for precise access to the surgical area for surgery, preservation of the entire posterior paravertebral muscle tissue, and minimalization of bone trauma [18]. In this study, the VAS-LBP score 1 week after surgery in the FE group was lower than that in the microscopical group (2.80 ± 0.92 vs 3.40 ± 0.50, *P* < 0.05). This difference may be attributable to the length of surgical incision and surgical method in the microscopical group. Paravertebral muscles and surrounding soft tissues are wounded as a consequence of the device’s dislocation, pulling and stretching of local tissues. Early functional exercise is better facilitated by FE microscopy, which can also help patients with early low back pain. However, the two groups did not vary significantly in terms of the pain reduction they experienced from lumbago and lower limb pain (*P* > 0.05).

It is currently reported that most studies reporting the postoperative clinical effects of LSS are focused on improving postoperative radiative pain, whereas few studies report improvements in postoperative numbness [25–29]. Typically, patients experience significant pain relief after lumbar decompression surgery, but the sensation of numbness does not improve as much as they might expect. As evaluating subjective symptoms of numbness is challenging, in this study we attempted to quantify the degree of numbness in the lower limb using NRS, allowing patients to self-report their level of discomfort. In this study, lower limb numbness and pain scores were significantly reduced 3 months after surgery, but there were no significant changes during follow-up. The NRS score of 2 groups was significantly lower than that of control group (*P* < 0.05), but there was no statistical significance (*P* > 0.05). Consequently, both surgical procedures are capable of reducing lower limb numbness in patients to a similar extent. Compared with preoperative, the proportion of patients with residual postoperative lower limb numbness (NRS > 1) was more than that of lower limb pain (VAS > 1) and disability (68.47% vs 56.76%). There was more likelihood of persisting numbness after surgery than pain, according to our results. It is important to note that despite the reduction in lower extremity NRS scores following surgery, patients may still retain the perception that LSS symptoms have not improved because of the persistence of numbness, thereby reducing their satisfaction with the treatment. Studies have previously indicated that the initial postoperative improvement of lower limb numbness symptoms is the most evident, followed by a gradual decrease. This study’s findings are consistent with those of others that found it difficult to instantly [30, 31] alleviate the numbness that often followed lumbar surgery. The lower limb numbness score decreased significantly 3 months after surgery, but there was no significant change after surgery. Regarding this, we speculated that the rapid recovery of lower limb numbness in patients at some time after surgery might be related to the rescue of reversible nerve injury. A significant change in the NRS from 3 months to follow-up was observed in the residual numbness, however, which is primarily caused by reversible nerve damage. There is also the possibility that the numbness in the lower extremities may be due to a torn dural. In this study, FE found 3 cases of dural tears (1 of which developed hypertension after dural rupture and forced the termination of surgery; During the microscopically controlled group, one patient remained in bed for seven days postoperatively, and antibiotics were given in order to prevent intracranial infection and surgical incision. The following are the primary considerations behind our study of dural tears in the EF group:1. Surgical technique selection requires further research. There was one patient with L2/3 stenosis, and we chose to use the Delta channel technique for treatment. 2. Inadequate hemostasis under the microscope, leading to damage due to bleeding in the surgical field; 3. A patient experienced significant adhesion owing to conservative epidural steroid injection, which led to an inadvertent tear during dissection because of inadequate preoperative preparation. However, different from previous literature reports [32–35], residual numbness and decreased muscle strength were found in only 1 out of 4 patients with sac tear during postoperative follow-up. We speculate that this may have something to do with the low number of cases.

The FE group had a longer average surgical time (92 minutes) than the Micro group (75 minutes) and, like most spinal endoscopic procedures, had a higher learning curve [36, 37]. This may be due to the fact that endoscopic surgery has a limited field of vision and operating region, and that there are discrepancies between the real operation and the light field, both of which might pose difficulties for the surgeon. Because hemostasis is already challenging under endoscopy, any failure to achieve full clotting will have a significant impact on the surgical process and make the patient more uncomfortable. A dural sac tear might potentially result from an inadequate surgical field. The patient in this study had a dural tear, which was predominantly caused by the worsening of the surgical area owing to hemorrhage, which presented some challenges to the physician while exposing the nerve roots. Regarding endoscopic hemostasis, studies [38–40] have pointed out that, compared with hemostasis by pressure of high water pressure, it is better to adjust patients’ blood pressure, because high water pressure will make the operative field become chaotic, and may also cause patients’ intracranial pressure height. Contrary to what may be expected, the operational field remained calm and the hemostatic effect remained effective despite the high pressure water being applied for just a little time. There was 1 patient with increased intracranial pressure after surgery, which was due to the increased blood pressure caused by the tear of the posterior dural sac, while the other patients did not have the above situation. Therefore, we speculate that this may be related to the water flow outside the surgical area and the control time of high pressure water compression. All patients were treated with high-pressure water to stop bleeding for no more than 30 seconds. Meanwhile, the anesthesiologist was asked to reduce the blood pressure to systolic pressure (120-100) mmHg and diastolic pressure (90-70) mmHg for hemostasis.

Although the operative time of FE group was significantly prolonged, the operative time was progressively shortened with the improvement of the operator’s proficiency, and the clinical effect was good without major surgical complications.

### Limitations

This study has several limitations. First, a modest number of instances were collected for this study sample. Secondly, the follow-up indexes changed greatly ranging from 1 week to 3 months demonstrated substantial variation. The outcomes of the experiments may have been different if more thorough follow-up had been conducted. We believe that the inaccuracy of findings can be mitigated if the follow-up time is more comprehensive. Third, statistical indications such C-reactive protein, creatine phosphokinase, etc. are inadequate when only lumbar pain VAS score, operation time, intraoperative blood loss are employed to evaluate surgical trauma during it weakens the evaluation’s scientific rigour by decreasing the number of quantitative indicators used. Fourth, for numbness symptoms, we attempted to utilize NRS for quantification, but “numbness” involved multiple symptoms, such as numbness, hypoesthesia, paresthesia and sensory disturbance, etc., the use of an 11-point numerical rating scale (NRS) for quantification was too general to refine the above symptoms; similarly, the NRS score could only indicate the intensity of numbness and was incapable of assessing the area. Lower limb numbness improvement can be overestimated when measured by NRS due to a particular bias compounded by the fact that patients’ numbness areas may diminish without a corresponding increase in numbness intensity. Fifth, this study’s follow-up time is brief to draw any conclusions about the treatment’s long-term efficacy.

## Conclusion

Positive outcomes from LSS are achievable using both microscope-assisted laminar fenestration decompression and Delta large-channel techniques. Nonetheless, there are some potential advantages to utilizing Delta large channels, including faster postoperative recovery, minor intraoperative trauma, and early alleviation from low back discomfort.

## Data Availability

Portions of the dataset used and/or analyzed in the current study are available from the corresponding author upon reasonable request.

## Abbreviations

LSS: Lumbar spinalstenosis
Delta: Inter laminar endoscopic surgical system,iLESSYS Delta
VAS: Visual Analogue Scale
ODI: The Oswestry Disability Index
BMI: Body Mass Index
VAS-LBP: Visual Analogue Scale-Low Back Pain
VAS-LBP: Visual Analogue Scale-Leg Pain
CT: Computed Tomography
MRI: Magnetic Resonance Imaging
SPSS: Statistical Package For Social Sciences.
